# Alterations of gut mycobiota profiles in intrahepatic cholangiocarcinoma

**DOI:** 10.3389/fmicb.2022.1090392

**Published:** 2023-01-06

**Authors:** Lilong Zhang, Chen Chen, Dongqi Chai, Tianrui Kuang, Wenhong Deng, Weixing Wang

**Affiliations:** ^1^Department of General Surgery, Renmin Hospital of Wuhan University, Wuhan, Hubei, China; ^2^Central Laboratory, Renmin Hospital of Wuhan University, Wuhan, Hubei, China; ^3^Hubei Key Laboratory of Digestive System Disease, Wuhan, Hubei, China

**Keywords:** gut mycobiome, intrahepatic cholangiocarcinoma, ITS2 rDNA sequencing, dysbiosis, *Candida albicans*

## Abstract

**Objective:**

Intrahepatic cholangiocarcinoma (ICC) is a silent liver malignancy with an increasing incidence. Gut mycobiota plays a crucial role in benign liver diseases; however, its correlation with ICC remains elusive. This study aimed to elucidate fungal differences in patients with ICC compared to healthy controls.

**Methods:**

The 40 fecal samples from 23 ICC patients and 17 healthy controls were collected and analyzed using ITS2 rDNA sequencing. Obtaining the OTUs and combining effective grouping, we carried out the biodiversity and composition of the fungi, as well as FUNGuild functional annotation.

**Results:**

Our results revealed the presence of intestinal fungal dysbiosis with significant enrichment of opportunistic pathogenic fungi such as *Candida* and *C. albicans,* and significant depletion of the beneficial fungus *Saccharomyces cerevisiae* in ICC patients compared with healthy controls. Alpha-diversity analysis demonstrated that patients with ICC showed decreased fungal diversity compared to healthy controls. Beta diversity analysis indicated that the two groups exhibited significant segregated clustering. Besides, *C. albicans* was found to be significantly more abundant in the ICC patients with TNM stage III-IV than those with stage I-II. The FUNGuild functional classification predicted that pathotrophs were the most abundant taxon in the ICC group, well above their abundance in healthy controls.

**Conclusion:**

This study indicates that dysbiosis of the fecal mycobiome might be involved in ICC development. Further research into gut fungi may contribute to new therapeutic options for ICC patients.

## 1. Introduction

Intrahepatic cholangiocarcinoma (ICC) is the second most common liver cancer (Beal et al., 2018). The number of deaths associated with liver cancer is predicted to be approximately 30,000 in 2022. Of these, ICC causes roughly 20% of these deaths, with a 5-year survival rate of under 20% (Yao et al., 2016; Wang et al., 2022). The incidence of ICC has risen by more than 140% over the last four decades (Saha et al., 2016). ICC typically arises against a backdrop of chronic inflammation that results in cholestasis and cholangiocyte damage (Khan et al., 2019; Labib et al., 2019). Hepatolithiasis, fibropolycystic disease, sclerosing cholangitis, viral hepatitis, obesity-related steatohepatitis, diabetes mellitus, parasite infections, and carcinogen exposure are all recognized risk factors for ICC (Welzel et al., 2007a,b). However, in most patients with ICC, no predisposing factors are found (Chang et al., 2009; Tyson and El-Serag, 2011; Khan et al., 2019). The majority of patients arrive with advanced-stage disease; diagnosing early ICC remains a challenge as most patients with early-stage disease are asymptomatic (Saha et al., 2016). Surgical excision remains the only potentially curative treatment option for ICC patients, but it is indicated in only 20–30% of patients (Endo et al., 2008). Even after complete surgical resection, the 5-year overall survival rate is about 20–35% (El-Diwany et al., 2019). Unfortunately, the efficacy of comprehensive treatment for ICC is also extremely limited. Consequently, there is an urgent need to explore the novel mechanisms of ICC development.

Currently, some studies have highlighted the connection between ICC development and gut microbiota dysbiosis (Jia et al., 2020; Deng et al., 2022). For example, Jia et al. discovered that ICC patients had higher levels of alpha-diversities and beta-diversities and higher abundances of *Lactobacillus*, *Actinomyces*, *Peptostreptococcaceae*, and *Alloscardovia* compared with normal individuals (Jia et al., 2020). Next, Deng et al. found that a diagnostic model constructed by intestinal bacteria significantly differentiated among healthy controls, hepatocellular carcinoma patients, and ICC patients (Deng et al., 2022). However, the role of microbial components other than gut bacteria, such as fungi, in ICC has not been explored, in part because of their relatively low abundance and the lack of well-characterized reference genomes (Parfrey et al., 2011; Underhill and Iliev, 2014). Thus, nothing is known about their contributions to the development of the ICC.

High-throughput sequencing techniques with increased capacity have provided access to the non-bacterial components of the gut microbiome. Even though gut fungi were thought to represent less than 1% of all commensal microbiomes by genomic equivalent (Underhill and Iliev, 2014), recent research has confirmed their importance in the gut (Iliev and Leonardi, 2017; Paterson et al., 2017). It assumes a stable role in the establishment and maintenance of the host immune system (Iliev and Leonardi, 2017; Paterson et al., 2017). Recently, Aykut et al. made the groundbreaking discovery that fungi could move from the intestinal lumen to the pancreas and that pathogenic fungi stimulate pancreatic adenocarcinoma *via* activating the mannose-binding lectin/complement-3 pathway (Aykut et al., 2019). Our previous review also detailed the close relationship between intestinal fungi and cancers of the colorectum, cavity, stomach, esophagus, and pancreas (Zhang et al., 2022). Thus, it is necessary to perform further studies regarding the changes in the composition of fecal fungi in the progression from health to ICC, thereby providing a new theoretical basis for the prevention and treatment of ICC.

## 2. Materials and methods

### 2.1. Participant recruitment

The ICC patients and healthy controls were prospectively recruited at the Renmin Hospital of Wuhan University from June 2021 to February 2022. The Helsinki Declaration and the Rules of Good Clinical Practice were followed during the conduct of this study. It was approved by our hospital’s clinical research ethics committee (WDRY2021-KS029) and registered on the Chinese Clinical Trial Registry Platform. A formal informed consent form was signed by each participant. Through the use of medical records and in-person interviews, the demographic information and clinical traits of patients were acquired. The ICC patients were diagnosed by histopathological examination of specimens from surgical resection or percutaneous ultrasound-guided liver needle core biopsy. Mixed-type liver cancer (hepatocellular carcinoma and ICC) was excluded. ICC patients had not received any routine anti-tumor therapy before the collection of feces samples. The individuals who came to our hospital for a medical examination were chosen as the healthy controls. Participants with other malignancies, digestive disorders, autoimmune diseases, and acute or chronic infectious diseases were excluded. To exclude hepatolithiasis, fibropolycystic disease, primary sclerosing cholangitis, viral hepatitis, and parasitic infections from affecting the intestinal fungus of ICC patients, we excluded ICC patients with these factors (although the number of these patients was small). In addition, none of the included subjects reported the use of probiotics, prebiotics, antibiotics, antifungals, or laxatives within the previous 3 months.

### 2.2. Sample collection, DNA extraction, PCR amplification, and ITS2 sequencing

A fecal sample was obtained by the participants, transported to the lab within 2 h, and stored at-80°C until DNA was extracted. The CTAB method was used to extract DNA, and 1% agarose gels were used to measure the DNA concentration. The hypervariable ITS2 region of the ITS gene was amplified using the primers [ITS3-2024F (5’-GCATCGATGAAGAACGCAGC-3′) and ITS4-2409R (5′- TCCTCCGCTTATTGATATGC-3′)] with the barcode. Mix the same volume of 1 x loading buffer (containing SYB green) with the PCR products and operate electrophoresis on 2% agarose gels for detection. The PCR products in the mixture were then purified using Thermo Scientific’s GeneJET Gel Extraction Kit. Using the TruSeq^®^ DNA PCR-Free Sample Preparation Kit (Illumina, United States), sequencing libraries were made according to the manufacturer’s instructions, and index codes were added. To evaluate the library’s quality, Thermo Scientific’s Qubit@ 2.0 Fluorometer was used. Finally, the library was sequenced, and 250 bp paired-end reads were produced on an Illumina Novaseq 6,000 PE250 platform.

### 2.3. Bioinformatics analysis

The NCBI Sequence Read Archive database contained the raw sequencing information for all samples (PRJNA898483). Paired-end reads were assigned to samples according to their unique barcode and truncated by cutting off the primer sequence and barcode. Then, FLASH (V1.2.7)^1^ was utilized to merge paired-end reads (Magoč and Salzberg, 2011). Quality filtering on the raw tags was conducted to obtain the high-quality clean tags (Bokulich et al., 2013) based on the QIIME (V1.9.1)^2^ quality-controlled process (Caporaso et al., 2010). Clean Tags were compared with the Unite Database^3^ to detect chimera sequences, and then the chimera sequences were removed. Using the Uparse software (Uparse v7.0.1001;^4^ Edgar, 2013) to cluster all Effective Tags, the sequences with ≥97% similarity were assigned to the same OTUs (Operational Taxonomic Units). The sequence with the highest frequency of occurrence in OTUs was chosen as the representative sequence of OTUs. For each representative sequence, the Unite Database (Kõljalg et al., 2013) was used based on the blast algorithm to annotate taxonomic information (Altschul et al., 1990).

A Venn diagram was produced *via* the R package “VennDiagram” (Version 2.15.3) to show the shared and unique OTUs between the two groups. Using R software (Version 2.15.3), the rank abundance curves were presented. Using QIIME (Version 1.9.1), the alpha diversity (Shannon, Simpson, Chao1, and ACE) and the beta diversity on weighted unifrac distances were determined. The non-metric multidimensional scaling (NMDS) analysis was conducted using the vegan package of R software (Kruskal, 1964), and the principal co-ordinates analysis (PCoA) analysis was completed using the WGCNA, stats, and ggplot2 packages of R software (Minchin, 1987). The R package vegan’s MRPP function was used for multiple response permutation procedure (MRPP) analysis (O'Reilly and Mielke, 1980). The linear discriminant analysis effect size (LEfSe) analysis was carried out using the LEfSe software (Version 1.0) with a default setting of 4 for the linear discriminant analysis (LDA) score screening (Segata et al., 2011). Spearman analysis of the top 20 species in abundance at the genus level with clinical parameters of ICC patients. To further explore the impact of mycobiota community change, functional prediction with FUNGuild annotation tools was deployed (A NHN, B ZS, B STB, 2016).

### 2.4. Statistical analysis

The categorical variables were compared using Fisher’s exact test. The Wilcoxon rank-sum test was used to compare continuous variables between the two groups. A two-sided *p*-value <0.05 indicated a significant difference.

## 3. Results

### 3.1. Characteristics of the participants

After a rigorous pathological diagnosis and exclusion process, 40 stool samples from 23 ICC patients and 17 healthy controls were collected and analyzed using ITS2 rDNA sequencing. Benefiting from a uniform sample collection protocol, all fecal samples were yellow and soft. The clinical characteristics of the participants, such as age, gender, and body mass index (BMI), were similar between the two groups (Table 1). Serum levels of albumin and platelets were significantly reduced in ICC patients compared with healthy controls (Table 1), while the remaining serum parameters were not significantly different (Table 1).

**Table 1:** Clinical characteristics of the enrolled participants.

**Parameter**	**Healthy controls (*n* = 17)**	**ICC patients (*n* = 23)**	***p-*values**
Age (year)	58.0 (46.1–64.1)	59.0 (49.0–67.0)	0.556
BMI (kg/m^2^)	22.3 (20.7–25.3)	22.6 (19.2–24.9)	0.432
Gender			
Female	3 (16.67%)	7 (20.59%)	0.470
Male	14 (83.33%)	16 (79.41%)	
ALT (9-50 U/l)	24.0 (17.6–31.6)	19.0 (14.0–33.0)	0.481
AST (15-40 U/l)	25.6 (22.0–30.6)	28.0 (21.0–50.0)	0.342
Albumin (40-55 g/l)	43.6 (42.0–47.7)	39.9 (36.6–43.6)	**0.001**
Globulin (20-40 g/l)	27.2 (22.4–30.9)	27.1 (25.2–33.6)	0.221
TBIL (0-23 μmol/l)	13.3 (12.6–19.3)	12.2 (10.8–21.4)	0.386
DBIL (0-8 μmol/l)	6.2 (4.2–7.2)	4.2 (3.5–7.0)	0.141
PT (9–13 s)	–	11.2 (10.6–12.5)	–
Platelet (125–350 10E9/L)	224.0 (202.4–252.4)	179.0 (119.0–220.0)	**0.004**
CA199 (0-37 U/ml)	–	22.9 (13.9–456.0)	–
CEA (0–5 ng/ml)	–	2.5 (1.5–4.0)	–
AFP (0–8.1 ng/ml)	–	3.4 (1.9–14.1)	–
Dietary habit	Mixed diet	Mixed diet	–

Median (interquartile range) or n (%); Continuous variables were compared using Wilcoxon rank-sum test between both groups. Categorical variables were compared using Fisher’s exact test. ICC, intrahepatic cholangiocarcinoma; BMI, body mass index; ALT, alanine aminotransferase; AST, aspartate aminotransferase; TBIL, total bilirubin; DBIL, direct bilirubin; PT, prothrombin time; AFP, alpha fetoprotein; CEA, carcinoembryonic antigen; CA199, carbohydrate antigen199. Bolded font represents *p*<0.05.

### 3.2. Patients with ICC display decreased fungal diversity compared to healthy controls

**Figure 1:**
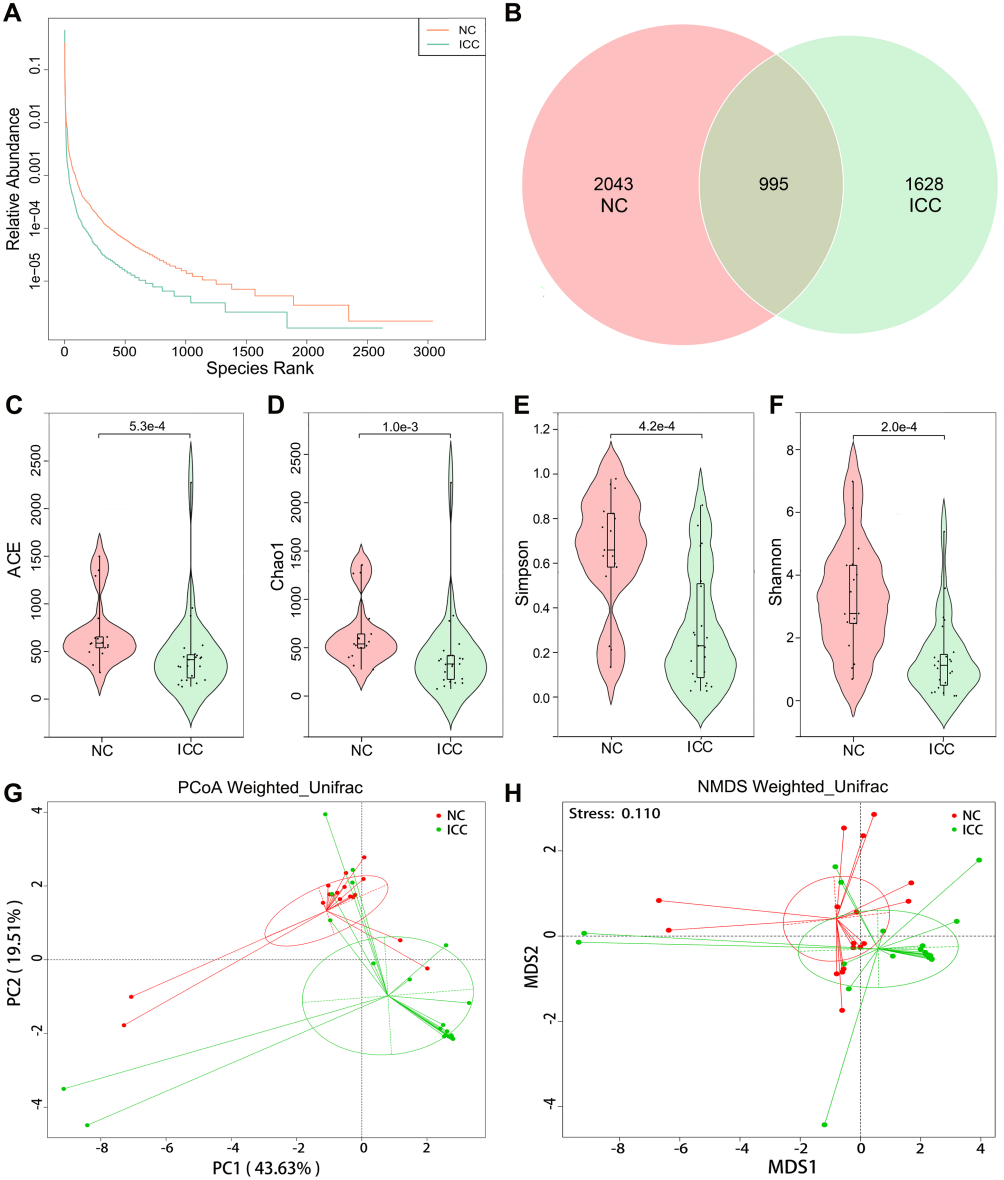
Diversity analysis in the ICC and NC groups. **(A)** Rank abundance curves. **(B)** Venn diagram displaying the overlap of OTUs identified between the two groups. Alpha diversity was estimated by the ACE index **(C)**, Chao1 index **(D)**, Simpson index **(E)**, and Shannon index **(F)**. The distributional difference of gut mycobiota profiles was assessed using PCoA **(G)** and NMDS (**H**) based on a weighted_unifrac matrix. ICC, intrahepatic cholangiocarcinoma; NC, healthy controls.

First, we explored the intestinal fungal diversity of the ICC patients and healthy volunteers. The rank abundance curves showed a good richness and uniformity of species in both groups (Figure 1A). The Venn diagram displayed that 995 OUTs were shared between the two groups. However, 2043 and 1,628 OTUs were unique to the control and ICC groups, respectively (Figure 1B). The community richness (Chao1 and ACE) and community diversity (Simpson and Shannon) indicators demonstrated a significantly decreasing tendency of mycobiota diversity in the ICC patients compared to healthy controls (ACE: *p* < 0.001, Chao1: *p* = 0.001, Simpson: *p* < 0.001, Shannon: *p* < 0.001; Figures 1C–F).

To display mycobiota space between the two groups, beta diversity analysis was conducted through PCoA and NMDS according to the weighted_unifrac matrix. The results indicated that the distribution of fecal fungal communities was significantly segregated clustering between the two populations (Figures 1G,H). Anosim and MRPP analyses further confirmed that inter-group differences were greater than intra-group differences and that there were significant differences in fungal community structure between the two groups (Anosim: *p* = 0.001, MRPP: *p* = 0.001; Supplementary Tables S1, S2). Therefore, these results suggest the presence of altered intestinal fungal composition in patients with ICC.

### 3.3. Differential mycobiota compositions between the ICC and healthy controls

**Figure 2:**
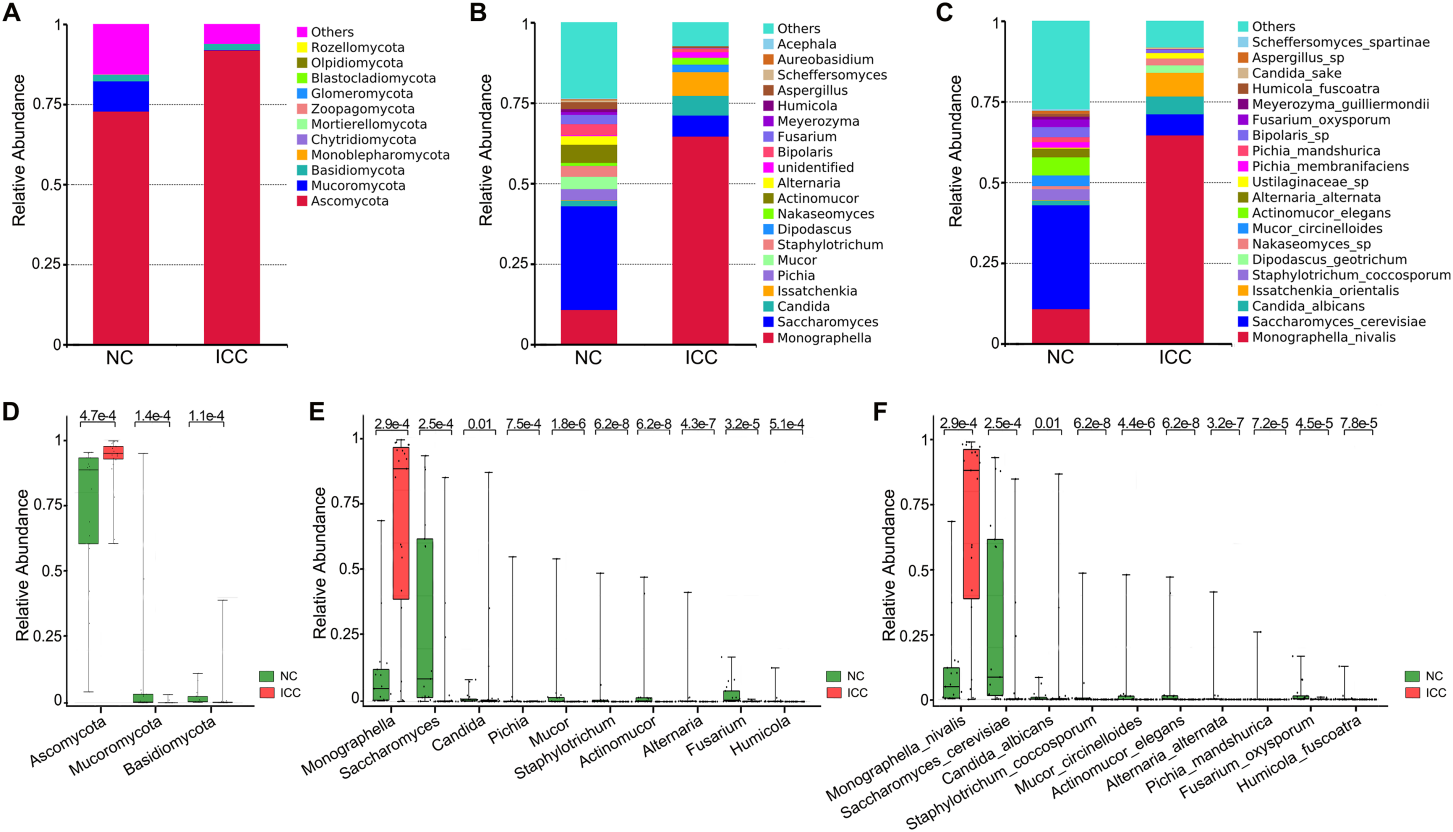
Differential analysis of fungal communities in the ICC and NC groups. Stacked bar plot of the predominant intestinal fungi at the phylum **(A)**, genus **(B)**, and species **(C)** levels. The three most abundant of the differential taxonomic units at the phylum level **(D)** between ICC patients and healthy controls. The ten most abundant of the differential taxonomic units at the genus level **(E)** and species level **(F)** between ICC patients and healthy controls. The Wilcoxon rank-sum test was used. ICC, intrahepatic cholangiocarcinoma; NC, healthy controls.

The stacked bar plot presented the distribution of the predominant fungi (top 20 most abundant taxa) at the phylum, genus, and species levels in participants from the two groups (Figures 2A–C). Next, the Wilcoxon rank-sum test was used to explore differences. The results showed that there were 6, 22, 56, 122, 190, and 249 significantly different taxa at the phylum, class, order, family, genus, and species levels between ICC patients and healthy controls, respectively (Supplementary Tables S3–S8). Compared to healthy controls, ICC patients had significantly higher relative abundances of *Ascomycota* and significantly lower relative abundances of *Mucoromycota* and *Basidiomycota* (Figure 2D; Supplementary Table S3). Besides, the 10 most abundant of the above differential taxonomic units at genus and species levels were presented in Figures 2E,F, finding a significant enrichment of *Monographella*, *M. nivalis*, *Candida*, *C. albicans*, *and depletion of Saccharomyces*, *S. cerevisiae*, *Pichia*, *P. mandshurica*, *Mucor*, *M. circinelloides*, *Staphylotrichum*, *S. coccosporum*, *Actinomucor*, *A. elegans*, *Alternaria*, *A. alternata*, *Fusarium*, *F. oxysporum*, *Humicola*, and *H. fuscoatra* in the ICC group.

### 3.4. ICC patients harbor a unique mycobiome signature compared to healthy controls.

**Figure 3:**
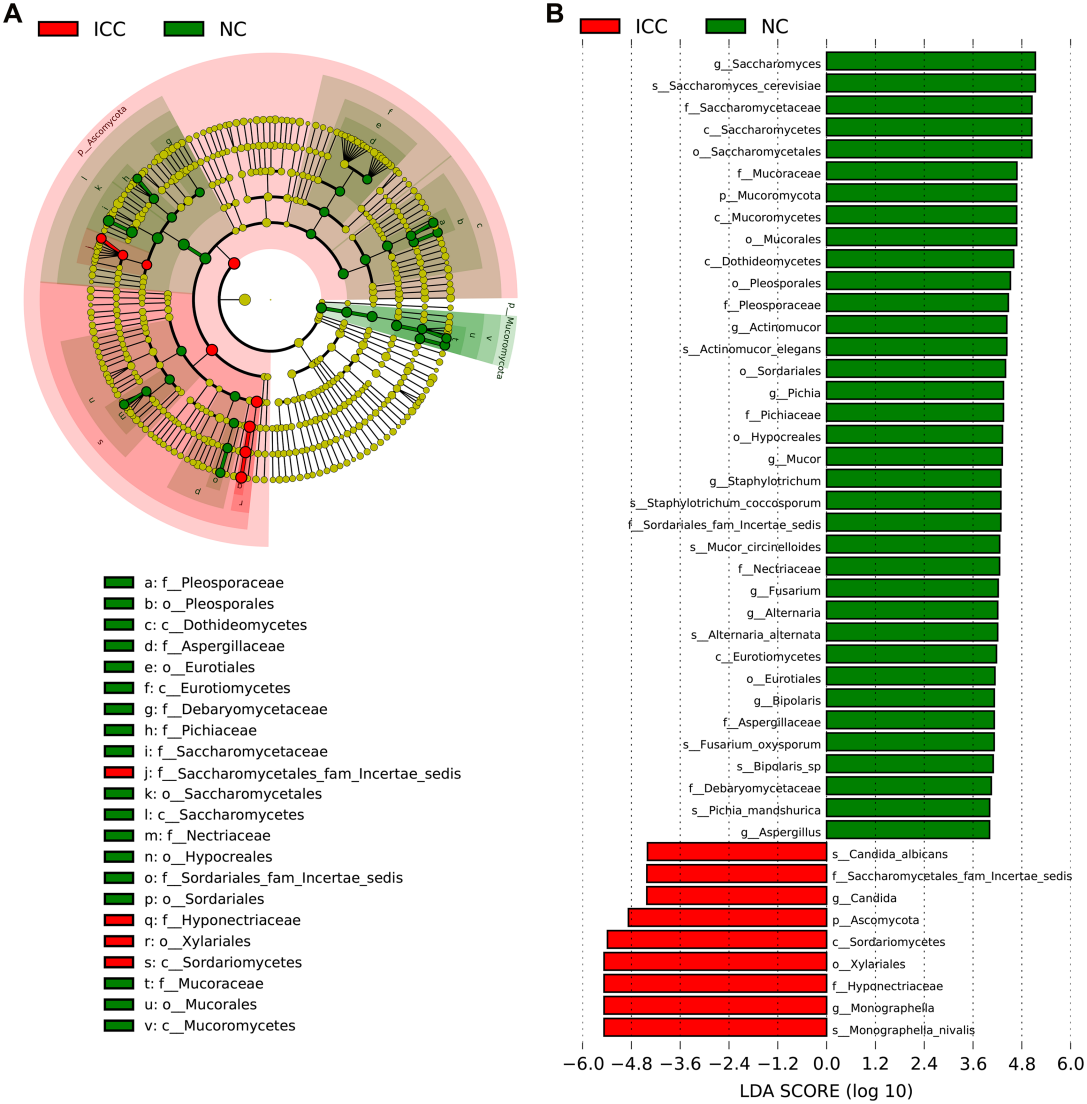
The differential taxa in the ICC and NC groups using the linear discriminant analysis effect size (LEfSe) analysis. **(A)** Taxonomic cladogram from LEfSe showing differences in fecal taxa of ICC patients and healthy controls. **(B)** LDA scores were computed for differentially abundant taxa in the gut fungi of ICC patients and healthy controls. Length indicates the effect size associated with a taxon. *p* = 0.05 for the Kruskal-Wallis sum-rank test; LDA score > 4; NC, healthy controls; ICC, intrahepatic cholangiocarcinoma; LDA, linear discriminant analysis.

The LEfSe with the criteria of an LDA score ≥ 4.0 was further used to identify differences in the fecal mycobiome between the ICC group and healthy controls. The predominant fungi and differential taxa between the two groups were displayed in the structure of the phylogenetic tree (Figure 3A). As shown in Figure 3B, the intestinal mycobiota of ICC patients were enriched with *Ascomycota*, *Saccharomycetes*, *Saccharomycetales fam Incertae sedis*, *Candida*, *C. albicans*, *Xylariales*, *Hyponectriaceae*, *Monographella*, and *M. nivalis*. For healthy controls, the results revealed significant up-regulation of the abundance of 36 taxa, such as *Saccharomyces*, *S. cerevisiae*, *Actinomucor*, *A. elegans*, *Staphylotrichum*, *S. coccosporum*, *Mucor*, *M. circinelloides*, *Alternaria*, *A. alternata*, *Fusarium*, *F. oxysporum*, *Pichia*, *P. mandshurica*, *Bipolaris*, and *Bipolaris* sp. These differentially abundant taxa can be considered potential biomarkers.

### 3.5. Correlation between gut mycobiota with TNM stage and clinical indicators in ICC patients

**Figure 4:**
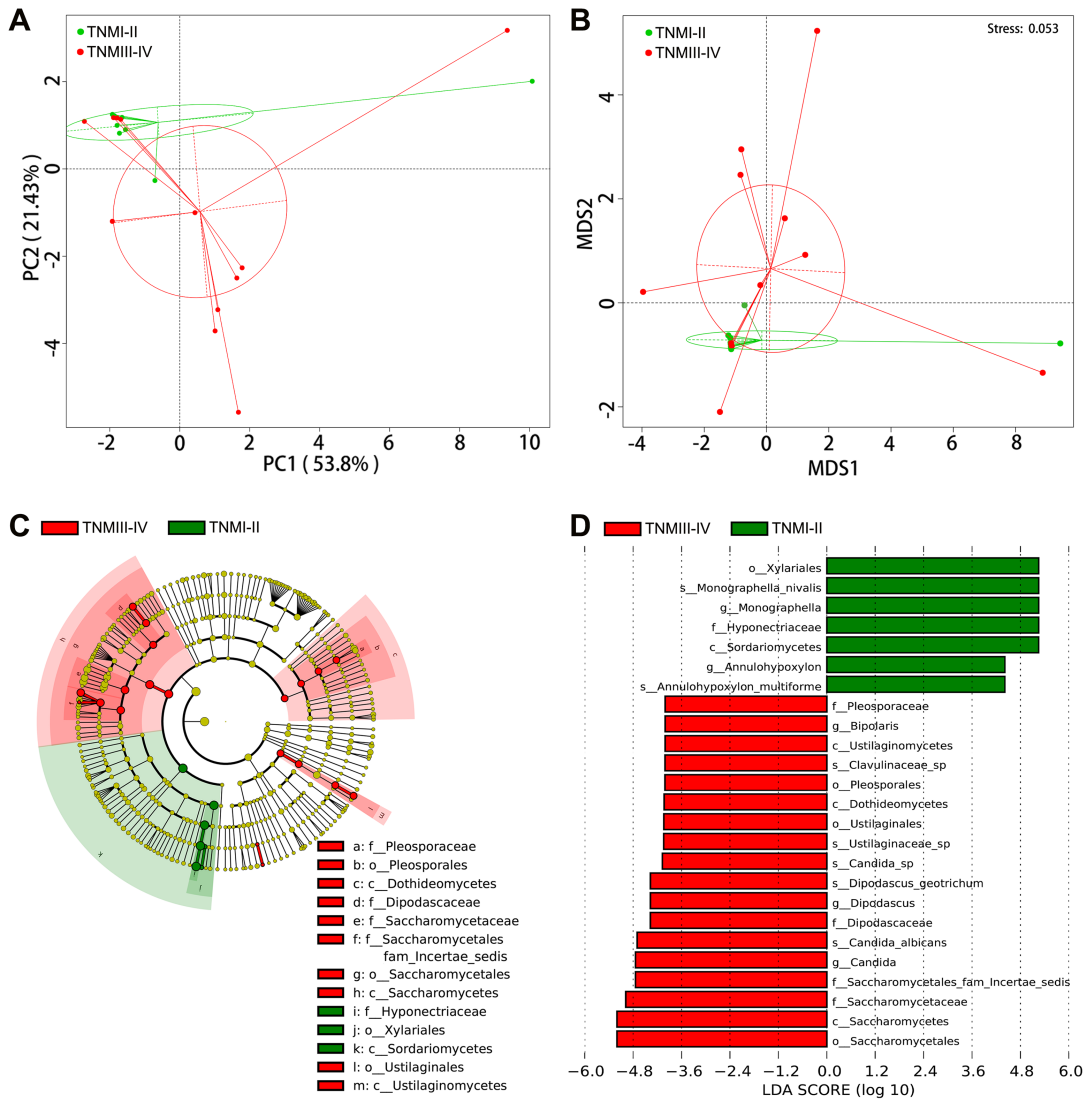
Correlation of gut mycobiota with TNM stage in ICC patients. The distributional difference of gut mycobiota profiles was assessed using PCoA **(A)** and NMDS **(B)** based on a weighted_unifrac matrix. **(C)** Taxonomic cladogram from LEfSe showing differences in fecal taxa of ICC patients with stage III-IV and stage I-II. **(D)** LDA scores were computed for differentially abundant taxa in the gut fungi of ICC patients with stage III-IV and stage I-II. Length indicates the effect size associated with a taxon. *p* = 0.05 for the Kruskal-Wallis sum-rank test; LDA score > 4; ICC, intrahepatic cholangiocarcinoma; LDA, linear discriminant analysis;

To further explore the correlation between fungal disorders and tumor progression, we further analyzed the differences in fungal taxa between 12 ICC patients with TNM stage III-IV and 11 ICC patients with TNM stage I-II (8th edition of the UICC/AJCC Cancer staging system). The baseline clinical data of the two groups is shown in Supplementary Table S9. We found that, compared to ICC patients with TNM stages I-II, the fungal flora of ICC patients with TNM stages III-IV exhibited significant segregated clustering, indicating that the gut fungal profile changes significantly with cancer progression (Figures 4A,B). The LEfSe method revealed that some fungal taxonomic units, such as *Candida*, *C. albicans*, *Candida sp.*, *Dipodascus*, *D. geotrichum*, *Ustilaginaceae sp.*, *Clavulinaceae sp.*, and *Bipolaris*, were significantly enriched in the ICC patients with stage III-IV (Figures 4C,D); however, *Sordariomycetes*, *Xylariales*, *Hyponectriaceae*, *Monographella*, *Monographella nivalis*, *Annulohypoxylon*, and *A. multiforme* significantly increased in the ICC patients with stage I-II. Thus, these results suggest that the opportunistic pathogen *C. albicans* may be involved in the progression of ICC.

We also analyzed the correlation between gut fungal microbes and clinical indicators. We found a significant positive correlation between *Dipodascus* and carcinoembryonic antigen (CEA), while *Saccharomyces* and *Rhizopus* showed a significant negative correlation with alpha-fetoprotein (AFP; Supplementary Figure S2). *Wickerhamomyces* was found to be significantly negatively correlated with alanine aminotransferase (ALT), and *Blumeria* and *Saccharomycopsis* were found to be significantly positively correlated with albumin (Supplementary Figure S2).

### 3.6. Functional classification prediction of the specific taxonomic

**Figure 5:**
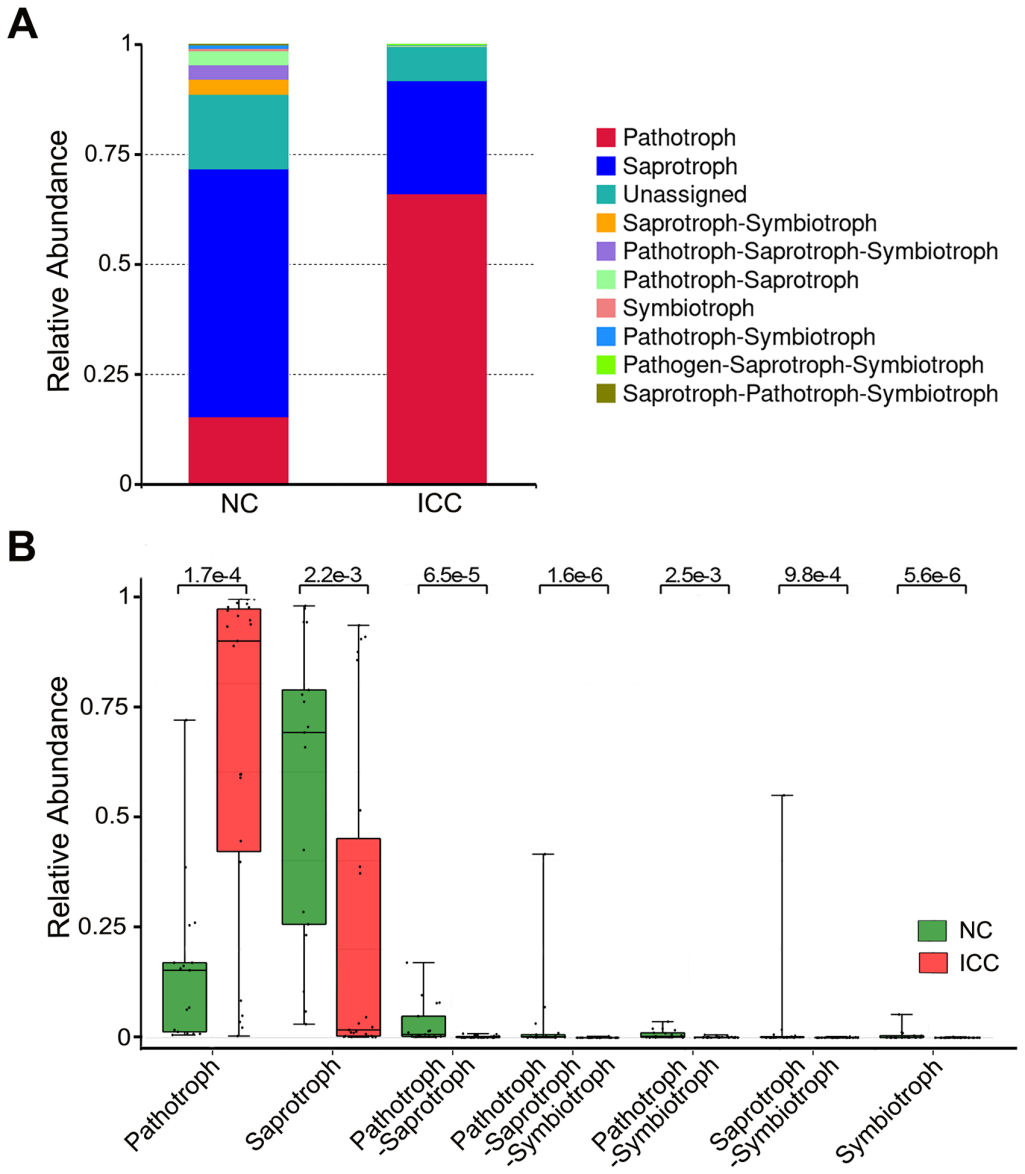
Functional predictive analysis between the ICC and NC groups. Stacked bar plot of the predominant functional classification at the trophic modes **(A)**. Wilcox rank-sum test was applied to the guild annotated results **(B)**. Control, healthy controls; ICC, intrahepatic cholangiocarcinoma.

Because of the lack of a powerful tool for annotating the function of fungi, we concentrated on the functional guilds of the fungal microorganisms instead, using FUNGuild. As is shown in Figure 5A, the stacked bar plot presented the distribution of the top 10 most abundant trophic modes among participants from the two groups. The most abundant mode in ICC patients was pathotroph, while the most common in healthy control patients was saprotroph (Figure 5A). Then, a Wilcox rank-sum test was implemented on the above modes. The results showed that pathotroph was significantly enriched in ICC patients, while saprotroph, pathotroph-saprotroph, pathotroph-saprotroph-symbiotroph, pathotroph-symbiotroph, saprotroph-symbiotroph, symbiotroph were significantly upregulated in healthy controls (Figure 5B). Thus, our results show that the symbiotic ecological associations of fecal fungi are changed in ICC patients and are dominated by pathological parasitism, which can receive nutrients from and adversely affect host cells.

## 4. Discussion

ICC is a silent liver malignancy with an increasing incidence and has been extensively studied to facilitate its diagnosis and management. In our study, we found for the first time that ICC patients had intestinal fungal dysbiosis, as evidenced by reduced alpha diversity, increased abundance of the opportunistic pathogenic fungi, *C. albicans,* and reduced abundance of the beneficial fungi, *S. cerevisiae*, compared to healthy controls. The FUNGuild functional classification predicted that pathotrophs were the most abundant taxon in the ICC group, well above their abundance in healthy controls. Besides, the abundance of *C. albicans* was remarkably higher in ICC patients with stage III-IV than in those with stage I-II. Our findings deepen our understanding of the association between the gut fungal microbiome and ICC, which has not been studied in the past.

The liver and the gut are closely linked anatomically and physiologically, and this ‘gut-liver axis’ could control liver pathology, intrahepatic and systemic immune responses. As such, the gut microbiota plays a significant role in modulating anti-tumor immunity (Gopalakrishnan et al., 2018; Matson et al., 2018; Routy et al., 2018). The gut barrier is the first line of defense to separate intestinal lumen microorganisms from the hosts (Peterson and Artis, 2014). Defects in the intestinal barrier function have been described in various liver diseases (Abu-Shanab and Quigley, 2010; Szabo, 2015). Impairment of barrier function increases intestinal permeability and facilitates the transport of microbial products and even intact organisms to portal circulation. In addition, Aykut et al. utilized *S. cerevisiae* labeled with a green fluorescent protein to treat tumor-bearing mice by oral gavage. They found that the fungus could migrate through the sphincter of Oddi, the pancreatic duct, and into the pancreas within 30 min (Aykut et al., 2019). Thus, gut fungi and their products may enter the liver *via* the portal vein and intra-and extra-hepatic bile duct routes. As we have found above, ICC patients have disorders of intestinal fungi. Dysfunctional fungal microbiota may be involved in the development of ICC through both of these pathways.

Interestingly, we also found that the abundance of *C. albicans* increased significantly in the ICC and that the alterations in *C. albicans became* more prominent as the TNM phase of the ICC progressed. Currently, *C. albicans* is by far the most studied opportunistic pathogenic fungus. The carcinogenic effect of *C. albicans* has long been disclosed in multiple cancers (Shiao et al., 2021; Wang et al., 2021; Zhong et al., 2021; Zhu et al., 2021; Liu et al., 2022; Vadovics et al., 2022). For example, Vadovics et al. (2022) revealed that *C. albicans* accelerated oral cancer development by upregulating the synthesis of matrix metalloproteinases, oncometabolite, and pro-tumor signaling pathways, as well as upregulating prognostic marker genes linked to metastatic events. Zhu and his colleagues (Zhu et al., 2021) reported that *C. albicans* can induce the upregulation of glycolysis in macrophages through the hypoxia-inducible factor (HIF)-1 pathway, thereby promoting the proliferation of intestinal epithelial cells and the progression of colitis-associated cancer (Zhu et al., 2021). Besides, although the prevalence of *M. nivalis* is significantly higher in ICC patients, no studies have been conducted to explore its correlation with the disease, and this needs to be further investigated.

In our study, *S. cerevisiae* and *A. alternata* were found to be depleted in ICC. *S. cerevisiae*, a major component of the human fecal mycobiome, has been demonstrated to lessen adherent-invasive *E. coli*-induced ileal colitis in a mouse model (Sivignon et al., 2015). Based on the *in vivo* studies, *S. cerevisiae* has also been shown to inhibit colorectal tumor growth by promoting epithelial cell apoptosis and modulating intestinal immunity and gut microbial structure (Li et al., 2020). Besides, secondary metabolites isolated from the endophytic fungus *A. alternata* showed significant anti-proliferative activity against human HCC cells (HUH-7) *in vitro* and *in vivo* (Palanichamy et al., 2019). Modes of action included cell cycle blockade and prevention of tumor growth (Palanichamy et al., 2019). The potential beneficial role of *S. cerevisiae* and *A. alternata* is highlighted by the decrease of *S. cerevisiae* and *A. alternata* found in ICC as found in this study. There is potential to intervene in this phenomenon as a therapeutic strategy for the prevention or treatment of ICC.

There are a few limitations to our study that should be mentioned. The small sample size included in this study did not allow the use of random forests to explore the diagnostic value of intestinal fungi for ICC. Besides, it is important to perform a complete characterization of the human intestinal microbiome because inter-kingdom interactions between the human bacteriome and mycobiome may unlock new pathways that could explain many unanswered questions. Finally, the carcinogenic effect of the fungus has yet to be confirmed in our subsequent animal studies.

## Data availability statement

The data presented in the study are deposited in the https://www.ncbi.nlm.nih.gov/ repository, accession number PRJNA898483.

## Ethics statement

The studies involving human participants were reviewed and approved by the Clinical Research Ethics Committee of the Renmin Hospital of Wuhan University. The patients/participants provided their written informed consent to participate in this study.

## Author contributions

LZ, WD, CC, and WW conceived and designed the study, and were involved in writing and revising the manuscript. LZ and CC were responsible for the collection of samples and clinical data. LZ, CC, DC, and TK were responsible for data analysis. All the work was performed under WD and WW instructions. All authors contributed to the article and approved the submitted version.

## Funding

This work was supported by grants from the National Natural Science Foundation of China (Nos. 82172855 and 81870442) and the Natural Science Foundation of Hubei Province, China (No. 2021CFB365).

## Conflict of interest

The authors declare that the research was conducted in the absence of any commercial or financial relationships that could be construed as a potential conflict of interest.

## Publisher’s note

All claims expressed in this article are solely those of the authors and do not necessarily represent those of their affiliated organizations, or those of the publisher, the editors and the reviewers. Any product that may be evaluated in this article, or claim that may be made by its manufacturer, is not guaranteed or endorsed by the publisher.

## Footnotes

^1^ http://ccb.jhu.edu/software/FLASH/

^2^ http://qiime.org/scripts/split_libraries_fastq.html

^3^ https://unite.ut.ee/

^4^ http://www.drive5.com/uparse/
