# Supplementary figures and images for: Alterations of gut mycobiota profiles in intrahepatic cholangiocarcinoma
Source: Front Microbiol. 2023 Jan 6;13:1090392. doi: 10.3389/fmicb.2022.1090392 (PMC9853418; doi:10.3389/fmicb.2022.1090392)

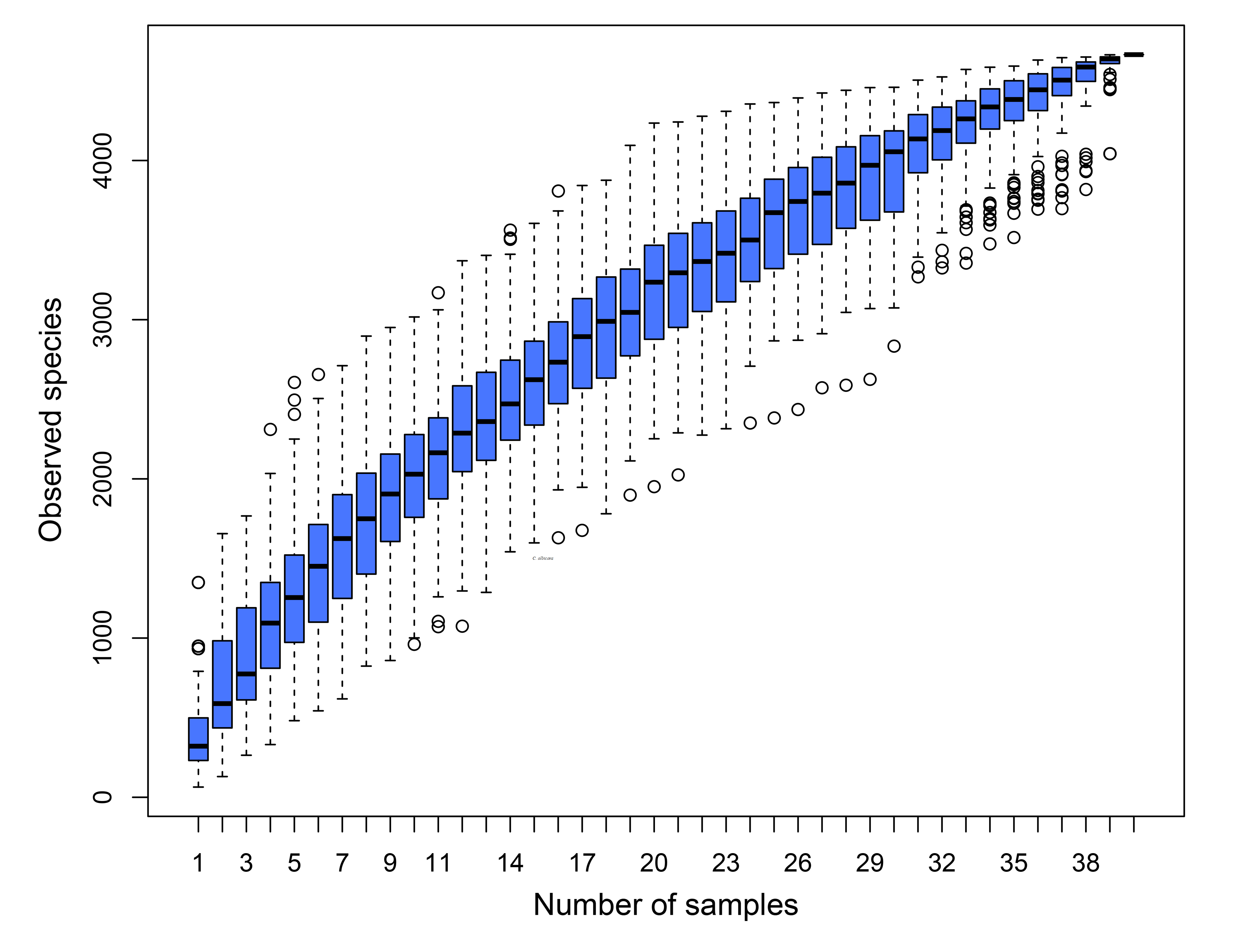

Supplement: Supplementary file 1 [file Image_1.TIF]

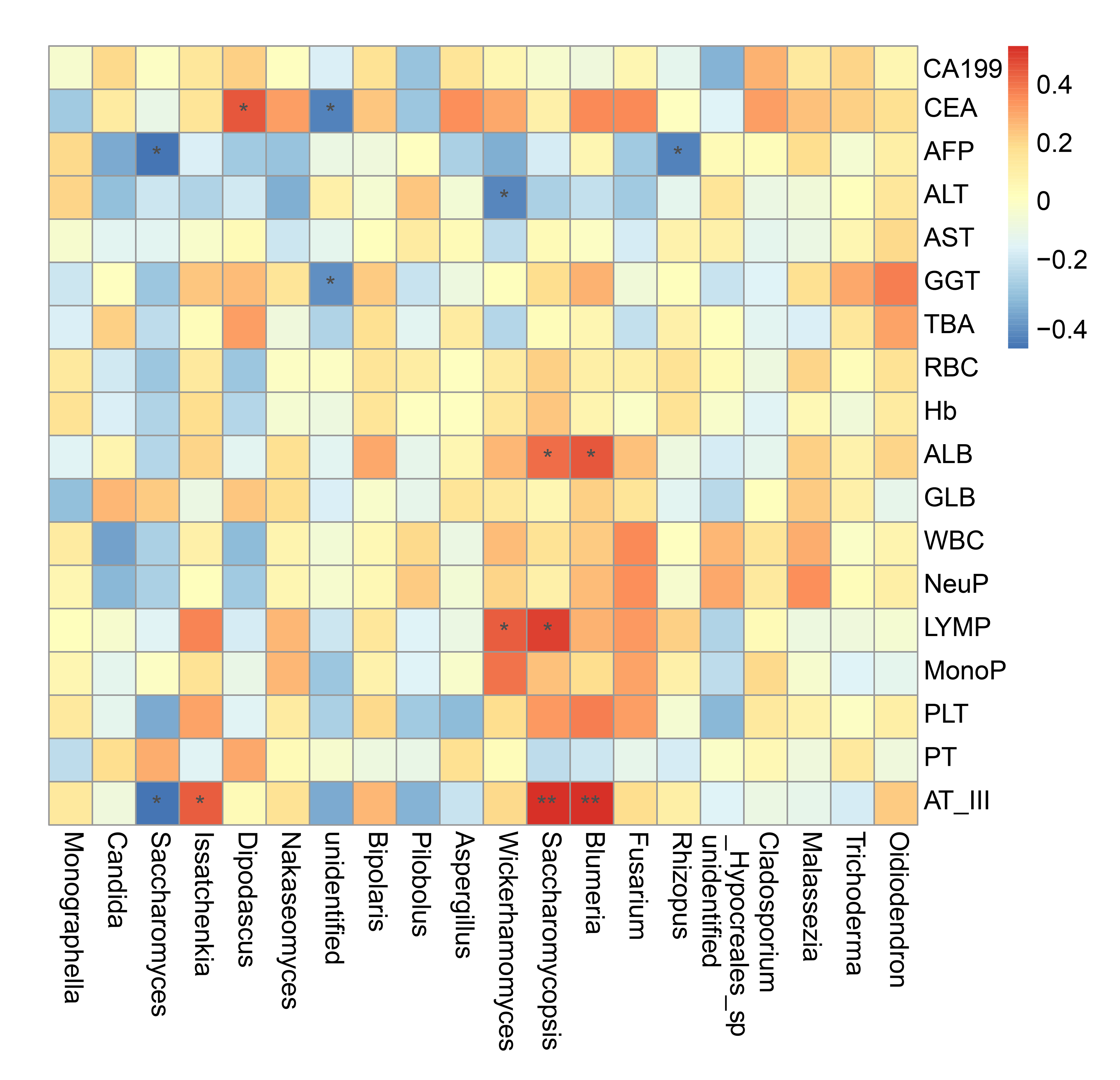

Supplement: Supplementary file 2 [file Image_2.TIF]
